# Supplementary material for: A neuroprotective tetrapeptide for treatment of acute traumatic brain injury
Source: EMBO Mol Med. 2025 Oct 1;17(11):3021–38. doi: 10.1038/s44321-025-00312-5 (PMC12603041; doi:10.1038/s44321-025-00312-5)
Supplement: Supplementary file 1 — Appendix [file 44321_2025_312_MOESM1_ESM.pdf]

Appendix

A neuroprotective tetrapeptide for treatment of acute traumatic brain injury

Aman P. Mann, Sazid Hussain, Pablo Scodeller, Hope N.B. Moore, Elan Sherazee, Rachel M. Russo, Erkki Ruoslahti

Table of Contents:

Appendix Figure S1. Nf-L levels in TBI mice after treatment......................................Page 2
Appendix Figure S2. Volcano plots of differentially expressed genes.........................Page 3
Appendix Table S1. Neuroscreen on mice with CCI. ....Page 4
Appendix Table S2. Safety study on CAQK in CCI mice. ....Page 5
Appendix Table S3. 7-Day IV dose study in rats. ....Page 6

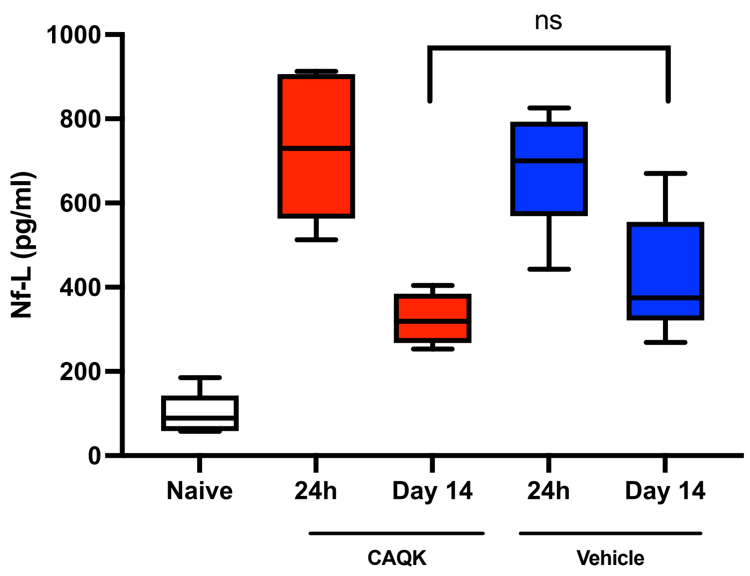

Appendix Figure S1. Nf-L levels in TBI mice after treatment. Serum levels of Nf-L in TBI mice at 24 hours and 14 days after brain injury plotted in a box and whiskers plot with whiskers down to the minimum, and up to the maximum, values. Data compared to naïve mice with no injury and no treatment. Plasma concentration shown (pg/ml; n= 5/group). Data were expressed as mean ± S.E.M. Differences were analyzed using unpaired t-test. n.s not significant.

A

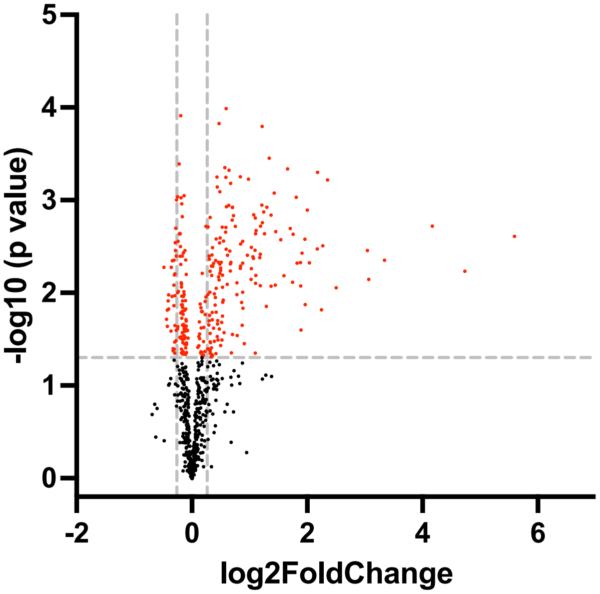

B

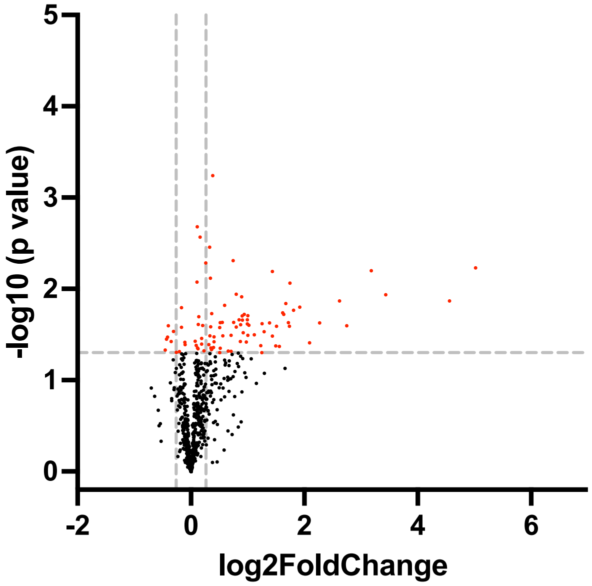

Appendix Figure S2. Volcano plots of differentially expressed genes. Differentially expressed genes that showed significance of ($p < 0.05$) compared between vehicle treated and naïve mice (A) and CAQK treated and naïve mice (B) using the neuropathology panel from Nanostring (n = 3. Generalized Linear Model (GLM) developed by NanoString used for calculating differential expression for count data.

	Naïve N=8	Vehicle N=8	CAQK N=8
General Health			
Poor coat condition	0	0	12.50%
Barbered hair	0	0	0
Missing whiskers	0	0	0
Piloerection	0	0	0
Unusual body tone	0	0	0
Unusual skin color	0	0	0
Unusual limb tone	0	0	0
Home cage behaviors			
Solitary sleeping	0	0	0
Fighting and aggression	0	0	0
Motoric, muscular abilities			
Positinal passivity	0	0	0
Trunk curl	0	0	0
Forepaw reaching	100.00%	100.00%	100.00%
Righting reflex	100.00%	100.00%	100.00%
Wire hanging	23.18	13.49	25.01
Reflexes			
Eye blink	62.50%	50.00%	37.50%
Ear twitch	50.00%	12.50%	37.50%
Whisker response	100.00%	25.00%	100.00%
Toe pinch response	75.00%	12.50%	75.00%
Reactivity			
Moving away on petting	100.00%	100.00%	100.00%
Struggling on restraint	100.00%	100.00%	100.00%
Vocalizing on restraint	50.00%	37.50%	25.00%
Dowel biting (3 pt. scale)	1.13	0.75	1.13
Empty cage behaviors			
Freezing on transfer	12.50%	0	12.50%
Wild running	0	0	0
Stereotypies	0	0	0
Cage exploration (3 pt. scale)	1.63	1.25	2

Appendix Table S1. **Neuroscreen on mice with CCI.** A neuroscreen was performed on mice at day 10 after CCI to assess for initial functional deficits. All data are expressed as a percentage of mice that displayed the phenotype shown, unless an alternative measure is stated in parentheses.

Groups	ALP (U/L)	ALT (U/L)	GGT (U/L)	TBIL (mg/dL)	BUN (mg/bL)
**1 mg/kg**	45 ± 5.3	24.3 ± 6.8	< 5	0.3	21.3 ± 3.5
**5 mg/kg**	47.4 ± 8.2	23.4 ± 4.9	< 5	0.3	22.2 ± 1
**25 mg/kg**	42.6 ± 7.6	34.8 ± 13.2	< 5	0.3	24.8 ± 2.05
**Vehicle; PBS**	45.7 ± 3.2	35.7 ± 10.8	< 5	0.3	21.3 ± 3.1

Appendix Table S2. Safety study in CCI mice. C57BL/6 mice (n = 5 per group) with CCI injury were intravenously injected daily for 7 days with vehicle control (PBS) or with the shown concentration of the CAQK peptide. After two weeks, blood was collected from the mice and analyzed for liver and kidney toxicity. Tests performed for liver function: ALP: Alkaline Phosphatase, ALT: Alanine Aminotransferase, TBIL- total bilirubin, GGT- G-glutamyl transferase, and for kidney function: BUN: Blood urea nitrogen. Results expressed as mean ± SD.

Appendix Table S3. 7-Day IV Dose Study in Rats. Sprague Dawley rats were randomized to 4 dose groups as shown in the table below. CAQK or vehicle (0.9% Sodium Chloride Injection, USP) was administered once daily by i.v. administration to male at 0
(vehicle),10, 100, or 300 mg/kg/day (Groups 1-4, respectively) for 7 consecutive days. The rats were euthanized and necropsied after blood sample collection for clinical pathology on Day 8.

A. Mean Red Blood Cell and Coagulation Parameters

Sex: Male		RBC (M/uL) [g]	HGB (g/dL) [g]	HCT (%) [g]	MCV (fL) [g]	MCH (pg) [g]	MCHC (g/dL) [g]	ABSRET (10^9/L) [g]
Group 1 - 0 mg/kg	Mean	7.003	14.87	43.20	61.67	21.20	34.47	297.80
	SD	0.295	0.21	3.47	2.42	0.70	2.57	95.55
	N	3	3	3	3	3	3	3
Group 2 - 10 mg/kg	Mean	7.077	14.50	44.13	62.33	20.50	32.90	374.87
	SD	0.125	0.53	1.11	0.81	0.36	0.61	43.35
	N	3	3	3	3	3	3	3
Group 3 - 100 mg/kg	Mean	7.160	14.33	43.43	60.70	20.03	33.03	354.37
	SD	0.572	0.95	2.75	1.31	0.49	0.12	9.68
	N	3	3	3	3	3	3	3
Group 4 - 300 mg/kg	Mean	7.070	14.70	42.63	60.37	21.00	34.67	345.93
	SD	0.791	0.35	3.82	2.05	2.33	2.80	22.21
	N	3	3	3	3	3	3	3

B. Mean White Blood Cell Parameters

Sex: Male		WBC (K/uL) [g]	ABNEUT (K/uL) [g]	ABLYMP (K/uL) [g]	ABMONO (K/uL) [g]	ABEOS (K/uL) [g]	ABBAS (K/uL) [g1]
Group 1 - 0 mg/kg	Mean	5.713	0.650	4.853	0.130	0.050	0.013
	SD	2.827	0.202	2.542	0.046	0.036	0.006
	N	3	3	3	3	3	3
Group 2 - 10 mg/kg	Mean	5.910	0.910	4.733	0.180	0.043	0.010
	SD	0.262	0.295	0.123	0.030	0.015	0.000
	N	3	3	3	3	3	3
Group 3 - 100 mg/kg	Mean	7.010	1.037	5.633	0.213	0.063	0.017
	SD	0.972	0.159	0.886	0.122	0.012	0.012
	N	3	3	3	3	3	3
Group 4 - 300 mg/kg	Mean	6.247	0.963	5.073	0.130	0.047	0.010
	SD	1.619	0.172	1.492	0.040	0.015	0.000
	N	3	3	3	3	3	3

C. Mean Serum Chemistry Parameters

Sex: Male		NA (mmol/L) [g]	K (mmol/L) [g]	CL (mmol/L) [g]	CA (mg/dL) [g]	PHOS (mg/dL) [g]	BUN (mg/dL) [g]	CREA (mg/dL) [g]
Group 1 - 0 mg/kg	Mean	141.7	5.47	106.3	10.07	9.10	11.3	0.47
	SD	1.5	0.35	1.5	0.06	0.20	1.5	0.15
	N	3	3	3	3	3	3	3
Group 2 - 10 mg/kg	Mean	141.3	5.00	106.7	10.00	8.77	10.7	0.47
	SD	0.6	0.20	1.5	0.26	0.50	1.2	0.06
	N	3	3	3	3	3	3	3
Group 3 - 100 mg/kg	Mean	140.3	5.27	106.3	10.13	9.20	11.0	0.53
	SD	0.6	0.15	0.6	0.57	0.35	2.0	0.23
	N	3	3	3	3	3	3	3
Group 4 - 300 mg/kg	Mean	141.0	5.03	106.7	10.23	8.97	10.3	0.53
	SD	1.0	0.21	2.1	0.12	0.29	3.1	0.06
	N	3	3	3	3	3	3	3
