# Supplementary material for: A neuroprotective tetrapeptide for treatment of acute traumatic brain injury
Source: EMBO Mol Med. 2025 Oct 1;17(11):3021–38. doi: 10.1038/s44321-025-00312-5 (PMC12603041; doi:10.1038/s44321-025-00312-5)
Supplement: Supplementary file 3 — Expanded View Figures [file 44321_2025_312_MOESM3_ESM.pdf]

Expanded View Figures

A B

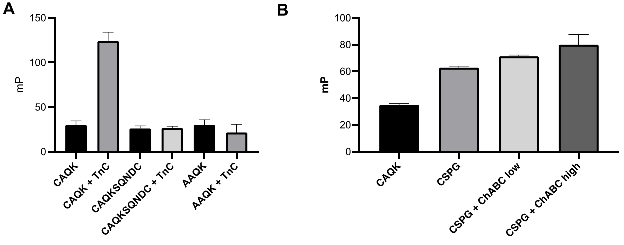

C

Chicken CSPG components	U251 ECM components
Neurocan	Laminin subunit alpha-5
Protein-tyrosine-phosphatase	Vimentin
Versican core protein	Galactin-3 binding protein
Aggrecan	Fibronectin
Brevican	Heat shock protein HSP 90-alpha
Agrin	Transitional endoplasmic reticulum ATPase
Tenascin-R	Tenascin-C
Decorin	Nidogen-1
Fibronectin	Filamin-A
Tenascin-C	Thrombospondin 1

D E

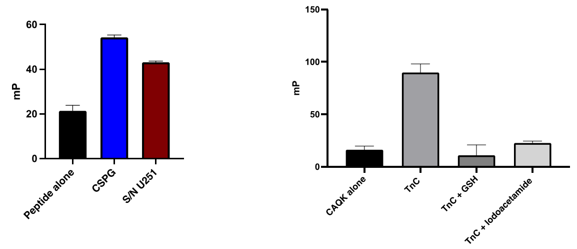

Figure EV1. CAQK binding to CSPG components.

(**A**) Fluorescence polarization (FP) measurement of CAQK binding to CSPG. FAM-CAQK (20 nM) was incubated with CSPG (200 nM) for 60 min at 37 °C. Binding with CSPG pretreated with chondroitinase ABC (chABC) at two concentrations low (5mU) and high (15mU) was compared to untreated CSPG. (**B**) FP assay to assess the binding of CAQK to brain ECM from different sources. FAM-labeled CAQK (20 nM) was incubated for 1 h at 37 °C with purified CSPG isolated from chicken brain (1 µM) or supernatant collected from cultured U251 human glioblastoma cell line. (**C**) Top hits from proteomic analysis of CSPG complex isolated from chicken brains and U251 conditioned media. (**D**) FP assay of binding of different peptides to TnC. FAM-labeled peptides (20 nM) were incubated with TnC (1 µM) for 1 h at 37 °C. (**E**) FP assay to assess the effect of the thiol group in cysteine of CAQK on binding to TnC. FAM-labeled CAQK (20 nM) was incubated with TnC (1 µM) for 1 h at 37 °C in the presence of GSH and Iodoacetamide. $n = 3$.

 © The Author(s)

A

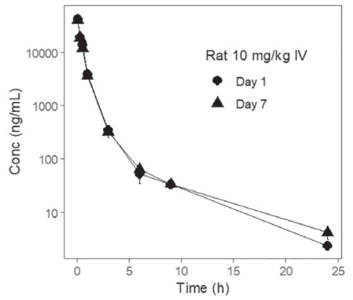

B

Parameter	Unit	Day 1	Day 7
k10	1/h	1.7	1.7
k12	1/h	0.074	0.090
k21	1/h	0.19	0.16
t1/2Alpha	h	0.39	0.38
t1/2Beta	h	3.9	4.6
C0	µg/mL	30.8	28.9
V1	L/kg	0.33	0.35
CL1	mL/min/kg	9.3	10.0
V2	L/kg	0.13	0.19
CL2	mL/min/kg	0.40	0.52
AUC 0-t	µg/mL*h	18.0	16.7
AUC 0-inf	µg/mL*h	18.0	16.7
MRT	h	0.82	0.90
Vss	L/kg	0.45	0.54

C

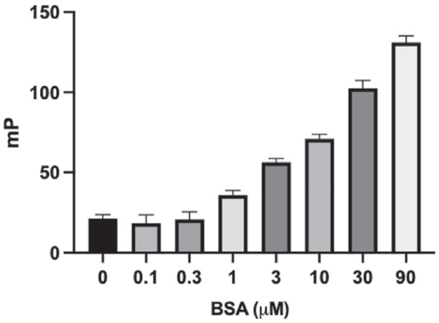

Figure EV2. CAQK plasma clearance in rats.

(**A**) CAQK was injected daily i.v. in healthy adult Sprague-Dawley rats at a 10 mg/kg dose for 7 days. Blood was collected at different time points at Day 1 and Day 7, and plasma was analyzed by LC/MS to analyze CAQK. Plasma concentration of CAQK was plotted. $N = 3$ per time point. Plasma clearance data were also used to calculate pharmacokinetic parameters in (**B**). (**C**) FP measurement of CAQK binding to BSA. FAM-CAQK (20 nM) was incubated with BSA at the indicated concentration for 1 h at 37 °C. $n = 3$.

© The Author(s)

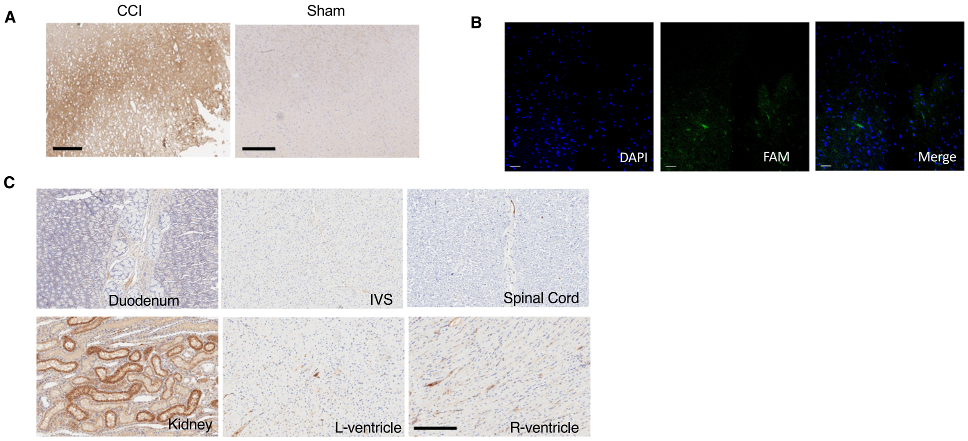

Figure EV3. CAQK targeting in the pig model of TBI.

(**A**) TnC expression is upregulated in pig CCI. Immunohistochemical staining for TnC on cortical pig brain sections shows elevated tenascin-C expression in the cortex surrounding CCI brain injury compared to the cortex of a sham-injured animal. Scale bar—300 µm. (**B**) Control peptide does not accumulate in the brain of a pig with CCI. Fluorescence imaging on cortical brain sections from CCI pig injected with FAM-AAQK. Sections were immunostained with anti-FAM (AAQK; green) and nuclei (blue). Scale bar—100 µm. (**C**) CAQK accumulation in different organs in pig CCI. A male Yorkshire pig with CCI was injected with FAM-CAQK at 6 h post-injury. IHC staining of FAM label on fixed paraffin-embedded tissue sections from different organs collected 1 h after peptide injection. Scale bar, 200 µm. IVS interventricular septum.

© The Author(s)
